# Supplementary material for: Impact of Powder Properties on the Rheological Behavior of Excipients
Source: Pharmaceutics. 2021 Aug 4;13(8):1198. doi: 10.3390/pharmaceutics13081198 (PMC8398033; doi:10.3390/pharmaceutics13081198)
Supplement: Supplementary file 1 [file pharmaceutics-13-01198-s001.zip › pharmaceutics-1300795-supplementary.pdf]

pharmaceutics

MDPI

Supplementary Materials: Impact of Powder Properties on the Rheological Behavior of Excipients

Pauline H. M. Janssen [1,*], Sébastien Depaifve [2], Aurélien Neveu [2], Filip Francqui [2], and Bastiaan H. J. Dickhoff [1]

Supplementary Figure S1. Scanning electron micrographs of the excipients used in this study: a) Lactopress® anhydrous, b) SuperTab® 21AN, c) SuperTab® 22AN, d) SuperTab® 24AN, e) Pharmatose® 80M, f) Pharmatose® 150M, g) Pharmatose® 200M, h) Pharmatose® 450M, i) SuperTab® 30GR, j) SuperTab® 11SD, k) SuperTab® 14SD, l) SuperTab® 50ODT, m) Pharmacel® 101, n) Pharmacel® 102, o) Pharmacel® sMCC90, p) Primojel®, q) Primellose®. The scale bars represent 100 µm.

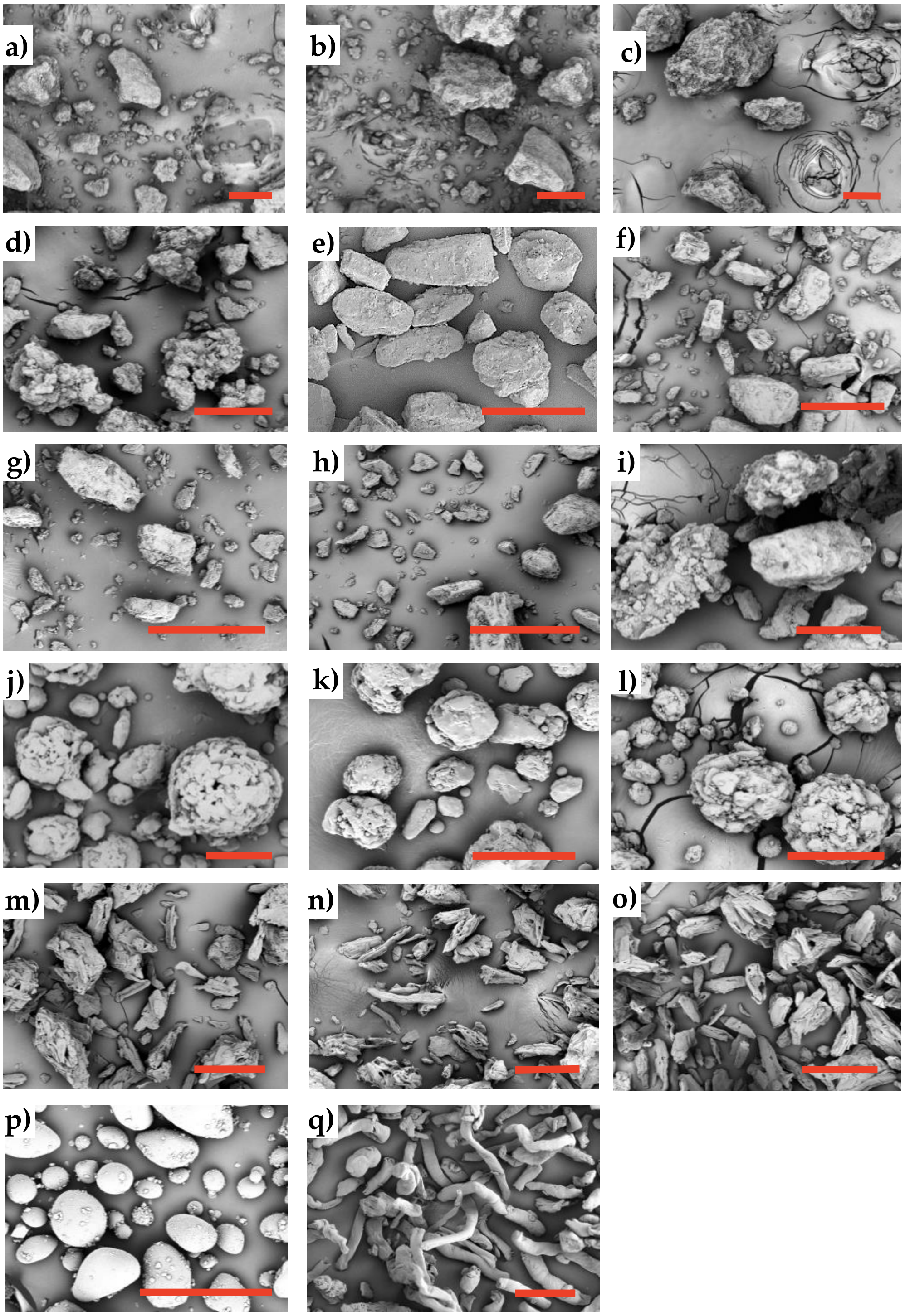

Supplementary Table S1: Physical properties for the set of excipients that is used in this study, being total moisture content by Karl-Fisher titration (KF), free moisture content by Loss On Drying (LOD), specific surface area (SSA), tapped density (TD), initial charge density (q_i), final charge density (q_f), tribo-charging density variation (Δq), true density (TrD), yield pressure at 0.01 mm/s (PyS), yield pressure at 300 mm/s (PyF) and strain rate sensitivity (SRS) .

Grade	KF (%w/w)	LOD (%w/w)	SSA (g/m²)	TD (g/mL)	q0 (nC/g)	qf (nC/g)	Δq (nC/g)	TrD (g/mL)	PyS (MPa)	PyF (MPa)	SRS (%)
Lactopress® anhydrous	0.1	0.1	0.3	0.89	-0.6	-3.6	-3.0	1.57	236	229	3
SuperTab® 21AN	0.3	0.0	0.4	0.92	-0.2	-2.6	-2.3	1.58	208	194	7
SuperTab® 22AN	0.2	0.1	0.4	0.80	-0.2	-2.6	-2.3	1.58	208	194	7
SuperTab® 24AN	0.8	0.1	0.5	0.68	0.0	-3.8	-3.8	1.54	166	155	7
Pharmatose® 80M	5.0	0.3	0.1	0.94	-0.3	-0.5	-0.2	1.54	171	172	-1
Pharmatose® 150M	4.7	0.3	0.4	0.98	-0.1	-0.1	0.0	1.53	185	175	6
Pharmatose® 200M	4.9	0.4	0.8	0.97	-1.5	-4.0	-2.5	1.54	189	186	2
Pharmatose® 450M	5.0	0.4	1.3	0.79	-1.2	-3.6	-2.4	1.54	194	173	12
SuperTab® 30GR	4.8	0.3	0.4	0.78	-0.4	-4.1	-3.7	1.53	192	175	10
SuperTab® 11SD	5.1	0.3	0.1	0.75	-0.1	-3.9	-3.7	1.54	170	154	11
SuperTab® 14SD	4.8	0.4	0.2	0.72	-0.8	-1.4	-0.6	1.53	184	165	11
SuperTab® 50ODT	4.7	0.1	0.2	0.83	-0.4	-3.3	-2.8	1.54	161	149	8
Pharmacel® 101	4.5	4.5	1.4	0.50	-0.5	-2.6	-2.1	1.54	81	79	2
Pharmacel® 102	4.2	4.2	1.2	0.49	-0.4	-3.5	-3.2	1.55	80	78	3
Pharmacel® sMCC90	4.5	4.5	5.1	0.50	-0.1	-2.1	-2.1	1.56	100	85	17
Primojel®	9.3	4.8	0.2	0.96	-0.3	-5.1	-4.8	1.52	145	98	48
Primellose®	9.3	5.3	0.3	0.74	0.2	-4.5	-4.7	1.53	158	121	31

Supplementary Table S2: Particle size distribution of the blends of different amounts of fines (Pharmatose® 450M) and coarse lactose (Pharmatose® 80M).

Amount of fines	x10 (µm)	x50 (µm)	x90 (µm)	Span
0%fines	49	238	374	1.37
5%fines	29	231	370	1.48
10%fines	20	223	365	1.55
15%fines	15	214	363	1.63
20%fines	12	202	360	1.72
25%fines	10	189	357	1.83
30%fines	8.7	173	353	1.99
35%fines	7.6	150	349	2.27
40%fines	6.7	109	344	3.09
60%fines	4.7	38	311	8.07
80%fines	3.7	27	238	8.84
100%fines	3.1	21	52	2.37
